# Effects of an Exercise and Lifestyle Education Program in Brazilians living with prediabetes or diabetes: study protocol for a multicenter randomized controlled trial

**DOI:** 10.1186/s13063-024-08535-6

**Published:** 2024-10-21

**Authors:** Lilian Pinto da Silva, Ana Paula Delgado Bomtempo Batalha, Gabriela Lima de Melo Ghisi, Mariana Balbi Seixas, Ligia Loiola Cisneros, Ann Kristine Jansen, Ana Paula Boroni Moreira, Daniele Sirineu Pereira, Raquel Rodrigues Britto, Danielle Aparecida Gomes Pereira, Patrícia Fernandes Trevizan, Paul Oh

**Affiliations:** 1 Faculty of Physical Therapy, Federal University of Juiz de Fora, Juiz de Fora, Brazil; 2 Graduate Program in Physical Education, Faculty of Physical Education and Sports, Federal University of Juiz de Fora, Juiz de Fora, Brazil; 3 KITE Research Institute, University Health Network, University of Toronto, Toronto, Canada; 4 Department of Physical Therapy, Federal University of Minas Gerais, Belo Horizonte, Brazil; 5 Department of Nutrition, Federal University of Minas Gerais, Belo Horizonte, Brazil; 6 Department of Nutrition, Federal University of Juiz de Fora, Juiz de Fora, Brazil; 7 Cardiovascular Rehabilitation and Prevention Program, Toronto Rehabilitation Institute, University Health Network, Toronto, Canada

**Keywords:** Diabetes mellitus, Prediabetes, Health education, Patient education, Exercise training, Randomized controlled trial

## Abstract

**Background:**

Patient education is a crucial strategy for promoting prevention and diabetes self-management since glycemic control achievement involves taking medications, medical nutrition therapy, physical exercise, and behavior changes. However, patient education programs are still barely implemented in low- and middle-income countries. This trial aims to investigate whether a lifestyle education intervention added to physical exercising is superior to sole physical exercising regarding functional capacity, disease-related knowledge, health behaviors, cardiometabolic health parameters, quality of life, depression, and diet quality in individuals with prediabetes or diabetes.

**Methods:**

Multicenter double-blinded randomized controlled trial with two parallel arms involving 12-week intervention and 6-month follow-up. The eligible individuals (≥ 18 years, living with prediabetes or diabetes, literate, no clinical decompensation and/or physical and/or mental limitations that contraindicate physical exercising, written physician permission for exercise, no cognitive impairment, no vision limitations for reading, no confirmed diagnosis of unstable coronary disease or heart failure, no pacemaker and/or implantable cardioverter-defibrillator, no complex ventricular arrhythmias, no intermittent claudication, no recent cardiovascular event or cardiac surgery, and no currently enrolled in a structured exercise program) were recruited from two Brazilian cities and randomized to either (1) an Exercise and Lifestyle Education Program (ExLE) or (2) an Exercise Program (Ex), which can be delivered on-site or remotely based on the participants’ internet access and technology literacy. The primary outcomes will be changes in functional capacity and disease-related knowledge. The secondary outcomes will involve changes in health behaviors (health literacy, physical activity level, exercise self-efficacy, and medication adherence) and cardiometabolic health parameters (glycemic control, anthropometric measures, and cardiac autonomic control). Program adherence, satisfaction with the program, diabetes-related morbidity, and changes in quality of life, depression, and diet quality will be the tertiary outcomes. Assessments will occur at baseline, post-intervention, and after 6-month follow-up.

**Discussion:**

If superior effectiveness of ExLE compared to Ex program to improve the outcomes measures is found, this program could be delivered broadly in the Brazilian health system, especially in the primary care facilities where most individuals living with prediabetes and diabetes in our country are assisted.

**Trial registration:**

ClinicalTrials.gov, NCT03914924. Registered on April 16, 2019.

**Supplementary Information:**

The online version contains supplementary material available at 10.1186/s13063-024-08535-6.

## Administrative information

Note: the numbers in curly brackets in this protocol refer to SPIRIT checklist item numbers. The order of the items has been modified to group similar items (see http://www.equator-network.org/reporting-guidelines/spirit-2013-statement-defining-standard-protocol-items-for-clinical-trials/).

**Table:** 

Title {1}	Effects of an Exercise and Lifestyle Education Program in Brazilians Living with Prediabetes or Diabetes: Study Protocol for a Multicenter Randomized Controlled Trial
Trial registration {2a and 2b}.	ClinicalTrials.gov, NCT03914924, registered on April 16, 2019 https://www.clinicaltrials.gov/study/NCT03914924
Protocol version {3}	Diabetes College Brazil Study, June 27, 2024
Funding {4}	APQ-02304-21 – Fundação de Amparo à Pesquisa do Estado de Minas Gerais
Author details {5a}	Lilian Pinto da Silva, PT, PhD ^1,2^ Ana Paula Delgado Bomtempo Batalha, PT, MSc ^2^ Gabriela Lima de Melo Ghisi,PT, PhD ^3^ Mariana Balbi Seixas, PT, PhD ^1^ Ligia Loiola Cisneros, PT, PhD ^4^ Ann Kristine Jansen, RD, PhD ^6^ Ana Paula Boroni Moreira, RD, PhD^7^ Daniele Sirineu Pereira, PT, PhD ^4^ Raquel Rodrigues Britto, PT, PhD ^4^ Danielle Aparecida Gomes Pereira, PT, PhD ^4^ Patrícia Fernandes Trevizan PT, PhD ^4^ Paul Oh, MD, MSc ^3,8^ ^1^Faculty of Physical Therapy, Federal University of Juiz de Fora, Juiz de Fora, Brazil ^2^Graduate Program in Physical Education, Faculty of Physical Education and Sports, Federal University of Juiz de Fora, Brazil^3^KITE Research Institute, University Health Network, University of Toronto, Toronto, Canada ^4^Department of Physical Therapy, Federal University of Minas Gerais, Belo Horizonte, Brazil ^5^ Department of Nutrition, Federal University of Minas Gerais, Belo Horizonte, Brazil ^7^ Department of Nutrition, Federal University of Juiz de Fora, Juiz de Fora, Brazil ^8^ Cardiovascular Rehabilitation and Prevention Program, Toronto Rehabilitation Institute, University Health Network, Toronto, Canada
Name and contact information for the trial sponsor {5b}	Lilian Pinto da Silva Faculty of Physical Therapy, Federal University of Juiz de Fora Av. Eugênio do Nascimento, s/n – Dom Bosco – CEP: 36038-330 - Juiz de Fora-MG, Brazil. E-mail: lilian.pinto@ufjf.br
Role of sponsor {5c}	The study sponsor, acting as the initiating study site, has overseen the study design, data collection, management, analysis, and interpretation of data. The preparation of the manuscript and the decision to submit it for publication were made by the sponsor. It is important to note that the funders played no role or had any authority in the study design, data collection, management, analysis, or interpretation of data. Furthermore, they will not be involved in the writing of associated publications or in the decision-making process regarding paper submission for publication.

## Introduction

### Background and rationale {6a}

While the global prevalence of diabetes continues to rise, there are indications that some high-income countries (HIC) are experiencing a stabilization or even a decline in new cases [1]. This trend is likely attributable to effective preventive measures and comprehensive public health education programs [2]. In contrast, diabetes rates among adults in Brazil are on a steady upward trajectory [3]. Additionally, the incidence of prediabetes is showing a marked increase on a global scale [4]. This contrast underscores the critical importance of sustained efforts in public health strategies tailored to different socioeconomic contexts.

The functional capacity and cardiac autonomic control are impaired in patients with diabetes [5, 6], and the former is related to poor prognosis due to increased cardiac risk and mortality [7, 8]. The mechanisms that explain the impact of diabetes on functional capacity are not completely known [6, 9]. The harmful effects of hyperglycemia on muscle strength and resistance, as well as other factors such as impaired glucose metabolism, long-term complications, and comorbidities, could contribute to the reduction of functional capacity in patients with diabetes [6, 9, 10]. Therefore, diabetes requires continuous and comprehensive medical care to prevent and manage its acute and long-term complications, which may affect the quality of life and patients’ daily physical capacity [11]. Diabetes treatment involves the use of medications and lifestyle modifications, including self-management education and support, medical nutrition therapy, and physical exercise to achieve glycemic control [12].

Although it is well known that physical exercise contributes to blood glucose control and the reduction of cardiovascular risk factors [11, 12], compliance remains a challenge. It demands overcoming several barriers to engage in a healthy lifestyle, such as being active [13]. According to data from the Surveillance System of Risk and Protection Factors for Chronic Diseases by Telephone Survey, adults with diabetes in Brazil do not get enough physical activity [14].

In this context, patient education is a fundamental component of diabetes care due to the effectiveness of educational interventions in promoting lifestyle change [12, 15], and the positive association between disease-related knowledge, treatment adherence, and a healthy lifestyle [16, 18]. Although diabetes guidelines recommend patient education as a component of diabetes care [12, 19], educational interventions for individuals living with or at risk of this condition have been scantly investigated in low- and middle-income countries (LMIC) such as Brazil [20, 21]. The impact of patient education on behavior change has not been frequently considered in these settings, with most studies on this topic being carried out in high-income countries [17, 22, 23]. Additionally, there is a lower adherence to self-care behaviors by patients with diabetes in LMIC compared to their peers in HIC which could be explained by the limited access to healthcare services [24].

A patient education program tailored to Brazilians living with diabetes and prediabetes was developed [25] drawing from the Diabetes College curriculum. Originally developed in English as part of the Diabetes Program at the Toronto Rehabilitation Institute in Canada, this curriculum has been validated by prior studies showing significant improvements in disease-related knowledge [26, 27], physical activity [26, 27], food intake [26, 27], exercise self-efficacy [26], and health literacy [26]. Given that physical exercise is recommended by the Brazilian guidelines to prevent and treat diabetes [28, 29], the patient education program for Brazilians was combined with an exercise program (Exercise and Lifestyle Education Program) to investigate whether patient education can promote better outcomes than physical exercise alone.

The feasibility, acceptability, and initial efficacy of the Exercise and Lifestyle Education Program were evaluated through a randomized pilot trial [30]. Additionally, its feasibility for remote delivery in an internet-based format was demonstrated [31]. Drawing from the results of the randomized pilot trial [30], we hypothesize that the Exercise and Lifestyle Education Program will yield significantly superior outcomes compared to exercise alone for individuals with prediabetes and diabetes in our setting.

### Objectives {7}

The purpose of this multicenter randomized controlled trial is to pragmatically investigate the effects of an Exercise and Lifestyle Education (ExLE) program compared to an Exercise Program (Ex) on functional capacity, disease-related knowledge, health behavior, and cardiometabolic health parameters in individuals with prediabetes and diabetes living in Brazil. Furthermore, program adherence, satisfaction with the program, quality of life, depression, diet quality, and 6-month related diabetes morbidity will also be investigated.

### Trial design {8}

This is a double-blinded (both outcomes’ assessors and data analysts) multicenter randomized controlled trial featuring two-arm parallel groups over 9 months (comprising a 12-week intervention period followed by a 6-month follow-up) with a 1:1 allocation ratio that follows the SPIRIT reporting guidelines [32].

## Methods: participants, interventions, and outcomes

### Study setting {9}

This study will be conducted in Juiz de Fora and Belo Horizonte, two cities in Minas Gerais, a southeastern Brazil’s state. Belo Horizonte, the state capital and largest city, is located in its Central Region, while Juiz de Fora, although medium-sized, holds the distinction of being the largest city in the Mata Region of Minas Gerais.

### Eligibility criteria {10}

Individuals from both sexes were eligible to participate in the study if they met all the following inclusion criteria and did not meet any exclusion criteria (Table 1).

**Table 1:** Inclusion and exclusion criteria

Inclusion criteria	
Age	≥ 18 years old
Current history of prediabetes or diabetes	Self-reported
Literate	Able to understand, read, and write a short simple statement on his/her everyday life
Written physician permission for exercising	Signed by the participant’s doctor
No cognitive impairment	Six-item screening scoring ≤ 4
No vision limitations for reading	
No confirmed diagnosis of unstable coronary artery disease or heart failure	
No pacemaker and/or implantable cardioverter defibrillator	
No recent cardiovascular event or cardiac surgery	≤ 6 months
No intermittent claudication	
No currently enrolled in a structured physical exercise program that follows diabetes guidelines	Accumulating 150 min per week of aerobic exercise and perform muscle-strengthening exercises two to three times per week
Exclusion criteria	
Clinical decompensation that contraindicates physical exercising	
Physical limitations	Any physical limitation that prevented the participant from engaging in the physical exercise
Mental limitation	Any limitation that prevented the participant to understand the educational content
Complex ventricular arrhythmias	Atrial flutter or atrial fibrillation, multiple atrial or ventricular ectopy, and atrioventricular or ventricular block

The potential trial participants were invited to self-respond to questions regarding inclusion criteria on a Google digital form. From the responses, those who seemed eligible were invited for the baseline assessment during which all the eligibility criteria were confirmed by the research team before starting the data collection. Cognitive impairment was assessed on-site by the six-item screener prior to collecting signatures on the informed consent form. The six-item screener is scored by a simple summation of errors, with individuals making more than two errors (i.e., scoring lower than four hits) identified as having cognitive impairment [33].

Participants who present cardiac electrical conduction or rhythm disturbances (e.g., atrial or ventricular ectopy and atrioventricular or ventricular block) or who have changes in cardiovascular medication prescription during the study will be excluded from cardiac autonomic control assessment.

### Informed consent {26a}

Research team members, previously trained in good clinical practices and about the study protocol, screened the eligibility of potential trial participants, enrolled those with confirmed eligibility, provided explanations of the study procedures, and collected signatures on the informed consent form from each participant.

### Additional consent provisions for collection and use of participant data and biological specimens {26b}

This trial will not collect biological specimens; therefore, biological specimens will not be stored or utilized for research purposes. All data collection procedures are outlined in the informed consent form.

## Interventions

### Explanation for the choice of comparators {6b}

Given the established evidence demonstrating the beneficial effects of physical exercise in managing blood glucose levels and reducing cardiovascular risk factors for individuals with diabetes [11, 12], as well as the national recommendations to exercise regularly to prevent and treat diabetes [28, 29], this study will employ an exercise intervention for the control group.

### Intervention description {11a}

The study interventions could be delivered on-site or remotely. Before randomization, the enrolled participants were screened for internet access and technology literacy using an instrument developed by the researchers based on best practices in digital health literacy [34] (see Additional file 1). Participants who answered “yes” to all questions were able to choose their preferred format for receiving the intervention. Those without internet access and/or limited technology literacy will receive the intervention on-site.

### Exercise Program

The Ex program was developed based on recommendations of the Brazilian Diabetes Society Guidelines [28, 29]. It will last 12 weeks and consist of aerobic exercise sessions designed to achieve a minimum of 150 min per week, with muscle-strengthening exercises incorporated two to three times per week starting from the fourth week of the intervention. Each exercise session will include warm-up (stretching), aerobic exercise (moderate-to-vigorous intensity walking according to the Borg Rating of Perceived Exertion exercise scale modified [35]), and muscle-strengthening using the own body weight and elastic bands (one-to-two sets of 10–12 repetitions of row, half-squat or leg extension, biceps curl, standing knee flexion, shoulder external rotation, heel raise, wall push up, lying down abdominal, elbow extension, and 15 s of plank).

For participants receiving the intervention on-site, 16 supervised 1-h exercise sessions will be delivered twice a week from the first 4 weeks and once a week from the fifth week onward. In addition, these participants will receive counseling to exercise in the community to accumulate 150 min per week of aerobic exercise and perform muscle-strengthening exercises 2 to 3 times per week.

Participants receiving the intervention remotely will first attend an on-site supervised exercise session to be instructed on the correct execution of aerobic and strengthening exercises to ensure proper performance. After this initial session, the exercise intervention will be delivered through a website specifically developed for the Ex program participants. Additionally, participants will receive weekly reminders about the exercise routine via WhatsApp messages from the research team.

Participants will receive guidance on recognizing signs and symptoms of effort intolerance and instructions on how to respond in such situations. They will be asked to record their weekly exercise routine and any exercise side effects in an exercise diary. Additionally, they will be encouraged to self-monitor their heart rate by taking their pulse before and after exercise. Participants who use insulin or secretagogue medications will be instructed to measure capillary blood glucose before and after exercise. Participants with hypertension will be counseled to measure blood pressure before exercise during the first two sessions or in subsequent sessions if symptoms suggestive of changing blood pressure levels are present.

### Exercise and Lifestyle Education Program

The ExLE program combines the Ex program with a patient education program tailored for Brazilians living with diabetes and prediabetes, with detailed descriptions available in a previously published study [25]. The ExLE will last 12 weeks and consist of the same procedures described for the Ex program plus eighteen education classes following the schedule of educational sessions for the Diabetes College program in Brazil [25].

For participants receiving the intervention on-site, 18 30-min education classes will be delivered twice a week during the first 4 weeks and once a week from the 5th week onward, before or after the supervised 1-h exercise sessions. Participants will receive a printed version of the Diabetes College patient guide [36].

Participants receiving the intervention remotely will first attend an on-site supervised exercise session to be instructed on aerobic and strengthening exercises exertion and ensure proper exercise execution. Additionally, they will receive orientation on navigating the ExLE program website to access and fill out exercise and study diaries, as well as access educational video lessons and other support materials. After this on-site session, both the exercise and patient education components of the program will be delivered through a website specific to ExLE program participants developed for this study.

The educational content will include:

1. Eighteen educational video lessons: recorded by the research team, lasting approximately 20 min each, and based on the Diabetes College program in Brazil;
2. Twelve videos related to weekly topics: THRiVE videos integrating chronic disease management and behavior change principles to help develop self-management skills through goal setting and action planning; and,
3. A printed version of the Diabetes College patient guide [36].

Additionally, participants will receive weekly WhatsApp text messages from the research team to remind them about the exercise routine, materials in their lesson plan, and the importance of tracking access to educational content in the study diary.

### Criteria for discontinuing or modifying allocated interventions {11b}

Since study participation is voluntary, participants have the right to withdraw from the study at any time. Upon withdrawal, no further data will be collected from them, although any data previously collected will be preserved in accordance with the terms outlined in the informed consent form. It is important to note that once a participant withdraws from the study, they will not be permitted to re-enter at a later date.

### Strategies to improve adherence to interventions {11c}

In on-site delivery mode, participants who did not attend the intervention session for that week will receive a WhatsApp message from the research team to remind them of the scheduled intervention for the following week. In remote delivery mode, the research team will check participants’ exercise and/or study diary completion to assess their engagement in exercise and/or education lessons. Participants who show no engagement in exercise and/or educational lessons for that week will receive a WhatsApp message reminding them about the exercise and/or study routine.

### Relevant concomitant care permitted or prohibited during the trial {11d}

All participants will receive counseling on exercising in the community to achieve the recommended 150 min per week of aerobic exercise and perform muscle-strengthening exercises two to three times per week using elastic bands provided during the intervention. Besides walking, participants may choose alternative aerobic exercise modalities such as swimming, running, or biking to meet the aerobic exercise recommendations.

### Provisions for post-trial care {30}

No post-trial care provision will be offered, as no harm is anticipated from trial participation, and participants will not receive compensation.

### Outcomes {12}

#### Primary outcomes

The primary outcomes of this study will include functional capacity and disease-related knowledge. Functional capacity will be measured by the distance covered in meters during the incremental shuttle walk test (ISWT) [37], while disease-related knowledge will be assessed using the total score of the Brazilian Portuguese version of the DiAbeTes Education Questionnaire (DATE-Q) [38]. The DATE-Q total score, ranging from 0 (indicating no disease-related knowledge) to 20 (indicating the highest level of disease-related knowledge) is obtained by a 20-item questionnaire with response options of true/false/do-not-know. The primary outcomes will be measured during baseline and outcomes assessment appointments.

#### Secondary outcomes

The secondary outcomes are divided in health behaviors (health literacy [39], physical activity level [40], exercise self-efficacy [41], adherence to Mediterranean diet [42], and medication adherence [43]) and cardiometabolic health parameters (glycemic control [44], anthropometric characteristics, and cardiac autonomic control [45]), as described in Table 2.

**Table 2:** Description of the secondary outcomes

Secondary outcomes	Measurement tools	Measurement description/range
Health literacy	NVS health literacy instrument	Total score ranging from 0 (no health literacy) to 6 (highest health literacy)
Physical activity level	Steps	Total steps/week and average of steps/day obtained from daily steps number for seven days measured by a pedometer
Exercise self-efficacy	Brazilian Portuguese version of BESES	Total score ranging from 0 (not confident) to 100 (completely confident)
Adherence to Mediterranean diet	Brazilian Portuguese version of the MDS	Total score ranging from 0 (no adherence) to 13 (highest adherence to Mediterranean food pattern)
Medication adherence	Brazilian version of the MAT ADOs and MAT insulin	Total score ranging from 0 (no adherence) to 7 (highest medication adherence)
Glycemic control	Results from A1c exams	Routine laboratory tests dated no longer than 3 months before the study assessment point
Anthropometric characteristics	BMI and waist circumference	Weight (kg) and height (m) measured to calculate BMI (kg/m^2^), waist circumference (cm) measured at the superior border of the iliac crest
Cardiac autonomic control	Five-minute resting HRV	SDNN (ms), RMSSD (ms), pNN50 (%), SD1 (ms), SD2 (ms), SD1/SD2 ratio, LF (ms^2^), HF (ms^2^), LF (nu), HF (nu), LF/HF ratio, TP (ms^2^)

*Abbreviations*: *NVS* Newest Vital Sign, *BESES* Bandura's Exercise Self-Efficacy Scale, *MDS* Mediterranean Diet Scale, *MAT ADOs* Brazilian version of the Measure of Adherence to Oral Antidiabetic, *MAT* insulin Insulin Treatments questionnaire, *A1c* glycated hemoglobin, *BMI* Body Mass Index, *HRV* Heart Rate Variability, *iNN* normal RR intervals, *ms* milissegundos, *SDNN* Standard deviation of iNN, *RMSSD* root mean square of the differences between successive iNN, *pNN50* percentage of successive iNN with a duration difference greater than 50ms, *SD1* Poincaré plot standard deviation perpendicular the line of identity, *SD2* Poincaré plot standard deviation along the line of identity, *LF* low-frequency spectral components, *HF* high-frequency spectral components,  *ms²* spectral components in absolute units, *nu*  spectral components in normalized units, *TP* total power

Health literacy, exercise self-efficacy, adherence to the Mediterranean diet, medication adherence, and all cardiometabolic health parameters will be measured during both the baseline and outcomes assessment appointments. Physical activity levels will be measured (1) over the 7 days following the baseline assessment appointment; (2) over the 7 days leading up to the post-intervention assessment appointment; and (3) over the 7 days following the post-follow-up assessment appointment or seven days leading up to this appointment to those participants who kept with them the pedometer worn in the past outcome assessment appointment.

#### Tertiary outcomes

The tertiary outcomes included in this study will be program adherence, satisfaction with the program, diabetes-related morbidity, quality of life, depression, and diet quality.

- Program adherence will be measured by the attendance rate in the program for each participant. The attendance rate of participants in the on-site delivery mode will be calculated based on the number of education classes and/or exercise sessions attended, divided by the total number of education classes and/or exercise sessions offered. For participants in the remote delivery mode, the attendance rate will be determined by the number of weeks exercise and /or study diaries were filled out, divided by the program’s duration in weeks.
- Satisfaction with the program will be measured by questionnaires developed by the researchers. For participants in the on-site delivery mode, the level of satisfaction with the patient education program will be assessed by an 11-item questionnaire, and with the exercise program by a 4-item questionnaire. For participants in the remote delivery mode, the level of satisfaction with the patient education program will be assessed by a ten-item questionnaire and with the exercise program by a five-item questionnaire.
- Diabetes-related morbidity will be assessed through the number and description of acute complications and diagnoses of chronic complications of diabetes, along with the number of hospitalizations associated with diabetes. The information will be collected using a three-item tool during the 6-month follow-up period.
- Quality of life will be evaluated using the Medical Outcomes Study 36-Item Short-Form Health Survey (SF-36) [46]. The total scores range from 0 (indicating no quality of life) to 100 (indicating the highest quality of life). This is obtained through a 36-item tool distributed across various physical and mental health domains, including physical functioning, social functioning, role–physical, bodily pain, mental health, role–emotional, vitality, and general health.
- Depression will be measured by the Brazilian version of the Center for Epidemiological Scale—Depression (CESD) [47]. The total score ranges from 0 (indicating no depressive symptoms) to 60 (indicating the highest level of depressive symptoms). This is obtained through a 20-item tool used to rate how often the interviewed experienced symptoms associated with depression over the past week, such as restless sleep, poor appetite, and feeling lonely. Response options range from 0 to 3 for each item (0 = rarely or none of the time, 1 = some or little of the time, 2 = moderately or much of the time, 3 = most or almost all the time).
- Diet quality will be measured through the consumption of macro and micronutrients. Food consumption will be assessed using an adapted version of a validated Food Frequency Questionnaire (FFQ) for Brazilian individuals with type 2 diabetes [48, 49], evaluating consumption frequency (daily, weekly, monthly, or yearly), number of portions, and serving size (small, medium, large or extra large). The adaptation of the FFQ was necessary since the individuals in this research have different eating habits than in the validated FFQ study, so regional adaptations were necessary. This adaptation was made based on a food diary on three non-consecutive days, including a weekend day carried out with 65 volunteers with type 1 or type 2 diabetes [42]. The final FFQ consists of 104 food items distributed across eight food groups (“cereals, tubers, roots, and derivatives”; “vegetables and legumes”; “fruits”; “beans”; “meat and eggs”; “milk and dairy products”; “oils and fats”; “sugars and sweets”), as well as 11 items investigating beverages, 4 items investigating oilseeds, and 1 item investigating food supplement.

### Participant timeline {13}

All assessment appointments will be scheduled individually, based on participants’ and research team members’ schedules.

After attending the baseline assessment appointment, the participant will be wearing a pedometer for 7 days before starting the intervention. To accommodate all participants enrolled at that time into the timetable of baseline assessment appointment and have their physical activity level measured before the intervention gets started, a period of up to 21 days including the 7-day wearing a pedometer will be allowed between the baseline assessment appointment and the first intervention session. Therefore, the baseline assessment could be carried out between one and 14 days previous to the first intervention session.

After the intervention conclusion, the participant will be wearing a pedometer for 7 days before attending the post-intervention assessment appointment. To accommodate all participants into the post-intervention assessment appointment timetable and have their physical activity level measured before this appointment, a period of up to 21 days including the 7-day wearing a pedometer will be allowed between the intervention conclusion and post-intervention assessment appointment. Therefore, the post-intervention assessment could be carried out between 7 and 21 days after the intervention conclusion.

Upon completing the 6-month follow-up, the participant will be wearing a pedometer for 7 days: (1) after attending the post-follow-up assessment appointment when this appointment is scheduled between 1 and 14 days after the 6-month follow-up completion or (2) before attending the post-follow-up assessment appointment when this appointment is scheduled between 7 and 21 days after 6-month follow-up completion. To accommodate all participants into the post-follow-up assessment appointment schedule and have their physical activity level measured after 6-month follow-up completion, a period of up to 21 days including the 7-day wearing a pedometer will be allowed between the 6-month follow-up completion and post-follow-up assessment appointment. Therefore, the post-follow-up assessment could be carried out between 1 and 14 days after the 6-month follow-up completion.

Table 3 shows the participant timeline.

**Table 3:**
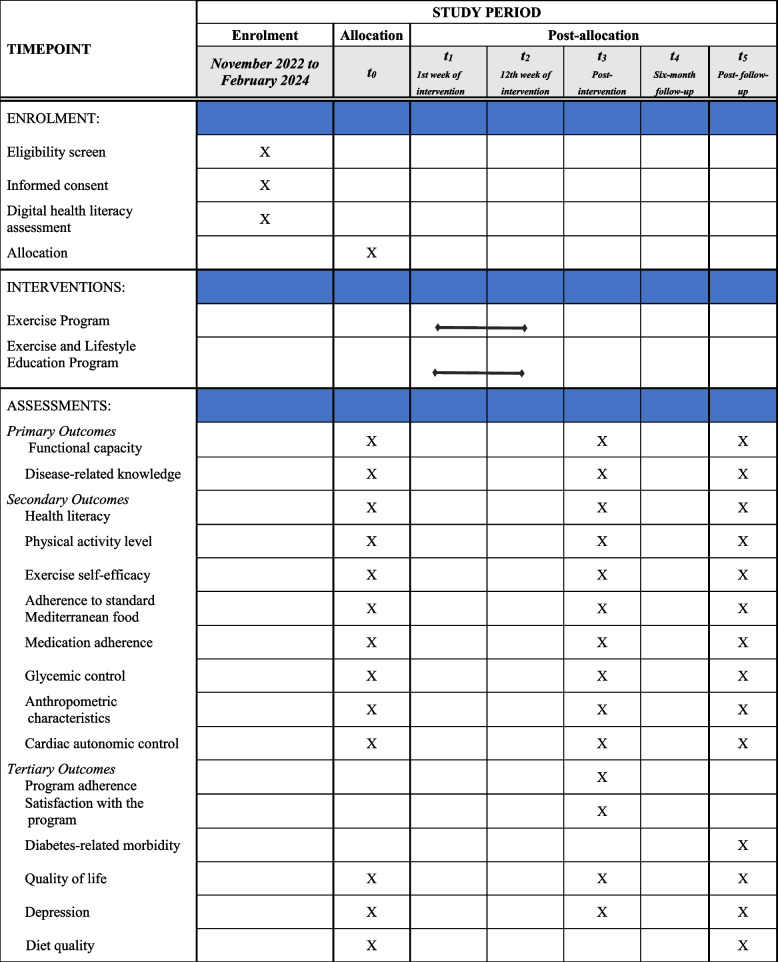
Overview of the enrollment, interventions and assessments during the trial

t0: 1 to 21-day interval pre-intervention for the baseline assessment, t3: 1 to 21-day interval post-intervention for the post-intervention assessment, t5: 1 to 21-day interval post-follow-up for the post-follow-up assessment

### Sample size {14}

The sample size was calculated considering the ISWT distance as the primary outcome, using parameters derived from a previous study involving educational intervention in cardiac rehabilitation [50]. The calculation was performed using R Software version 3.4.3, with the following parameters: a moderate effect size (*d* = 0.20), 80% statistical power, a 5% alpha level, one-sided test, two arms, and three measurements. A total sample size of 200 participants (100 per arm) was determined. Assuming a 20% attrition rate, based on a previous study [50], 120 participants would be enrolled in each arm to ensure that 200 participants complete the study protocol.

### Recruitment {15}

The research team recruited potential participants through face-to-face interactions at health services in the two cities, as well as phone calls to individuals listed in a database from previous studies conducted by the research group [38, 41, 42], none of which involved exercise or educational interventions. In addition, social media advertisements were utilized, and the study was disseminated among local healthcare providers through face-to-face interactions and via email among employees of the Federal University of Juiz de Fora (UFJF) and the Federal University of Minas Gerais (UFMG), respectively.

## Assignment of interventions: allocation

### Sequence generation {16a}

A block random assignment sequence was generated with a 1:1 ratio, using the website www.randomization.com by assigning enrolled individuals to one of the two study arms in each research center.

### Concealment mechanism {16b}

The principal investigator maintained an allocation sequence for each center in two separate password-protected files. It was shared with the research center coordinators, who distributed the allocated program to the intervention team members only after conducting theparticipants’ baseline assessment. This allocation information will not be accessible to the outcome assessors to ensure allocation concealment. 

### Implementation {16c}

After the baseline assessment, the center research coordinator received the participant’s allocation from the principal investigator. The allocation was disclosed to the participant and the intervention team members, but it remains concealed from the outcome assessors, who are blinded to the allocated intervention.

## Assignment of interventions: blinding

### Who will be blinded {17a}

Due to the nature of the intervention, participants and the intervention team members cannot be blinded to the allocation. However, to ensure objectivity, two independent research team members, blinded to the allocation, will handle database management and conduct statistical analyses, respectively.

### Procedure for unblinding if needed {17b}

Given that the trial does not involve drug testing, there will be no need for emergency unblinding.

## Data collection and management

### Plans for assessment and collection of outcomes {18a}

All research team members will receive training specific to their assigned responsibilities in the research, whether it involves conducting baseline assessments or outcome assessments. Additionally, they will adhere to a research-specific manual tailored to their respective roles to ensure that all procedures are carried out in accordance with the validation of assessment tools or any necessary training, especially for tools developed by the research team.

### Plans to promote participant retention and complete follow-up {18b}

There are no plans to promote participant retention. To minimize dropouts on the 6-month follow-up, the participants will receive monthly phone calls to remind them about filling out the diabetes-related morbidity tool and the post-follow-up assessment appointment.

### Data management {19}

All trial data will be entered into an Excel file by an independent research team member who is not an intervention team member, outcome assessor, or data analyst. Additionally, 30% of the entered data will be randomly checked by one of the research center coordinators.

### Confidentiality {27}

The collected data will be anonymized using individual participants’ coding to label the trial data. The link between identifiable personal data and the codes will be stored securely and separately from the trial data.

### Plans for collection, laboratory evaluation, and storage of biological specimens for genetic or molecular analysis in this trial/future use {33}

No biological specimens will be collected in this trial.

## Statistical methods

### Statistical methods for primary and secondary outcomes {20a}

The participants’ characteristics will be presented as central tendency and dispersion measures or absolute values and percentages. These data will be compared using the independent *t*-test for continuous variables and chi-square or Fischer’s exact test for categorical variables.

#### Primary and secondary outcomes

Primary and secondary outcomes will be presented as central tendency and dispersion measures. Data distribution will be analyzed using the Shapiro–Wilk test. The analysis of variance (2 × 2 ANOVA) will compare data between baseline and post-intervention and between groups. An alpha of 5% will be considered for statistical significance, and post hoc comparisons will be performed to detect two-by-two differences. In case of missing data, the statistical procedures will follow the intention to treat.

#### Tertiary outcomes

Quality of life, depression, and diet quality will be presented and analyzed as the primary and secondary outcomes. Data from the program adherence and morbidity associated with diabetes will be analyzed using the independent *t*-test. Regarding satisfaction with the program, it will be analyzed descriptively from absolute values and percentages.

### Interim analysis {21b}

There is no planned interim analysis.

### Methods for additional analysis {20b}

An interaction analysis to test the effect of the intervention delivery mode (on-site or remote) is planned.

### Methods in analysis to handle protocol non-adherence and any statistical methods to handle missing data {20c}

The value 0 (zero) will be imputed to replace the missing data in the analysis that will follow the intention-to-treat principle.

#### Plans to give access to the full protocol, participant-level data, and statistical code {31c}

The study plan is accessible to the public through the ClinicalTrials.gov register (NCT03914924). The data supporting the findings of this study will be available from the corresponding author upon reasonable request.

## Oversight and monitoring

### Composition of the coordinating center and trial steering committee {5d}

This study will be overseen by a principal investigator who will be responsible for supervising the research center coordinators and all trial-related activities. The principal investigator will ensure that trial objectives and targets are met according to the established study schedule and protocol, as well as allocate financial resources to the trial development. Research center coordinators, in turn, will be responsible for creating a schedule for recruitment, assessments, and interventions. They will also supervise all research team members’ activities, including the assessment and intervention teams. This trial does not have a trial steering committee with independent members, nor does it include stakeholder or public involvement groups.

### Composition of the data monitoring committee, its role, and reporting structure {21a}

The present study does not establish a data and safety monitoring board, as both the exercise and the education interventions are considered low-risk interventions with no substantial safety issues.

### Adverse event reporting and harms {22}

Due to the low-risk nature of the trial, no adverse event is anticipated. Any unintended effects of exercise intervention will be reported to the Research Ethics Committees at the Federal University of Juiz de Fora or the Federal University of Minas Gerais.

### Frequency and plans for auditing trial conduct {23}

A data monitoring committee was not deemed necessary in the randomized controlled trial due to the low risk of adverse events and the robustness of the study protocol ensuring participant safety and data integrity.

### Plans for communicating important protocol amendments to relevant parties (e.g., trial participants, ethical committees) {25}

Any revision to the trial will be reported to the ethics committee. After receiving the approval of the protocol revision, we will communicate it to the investigators.

### Dissemination plans {31a}

The results of this trial will be available through scientific publications in a peer-reviewed journal and at scientific conferences.

## Discussion

To our knowledge, this is the first randomized controlled trial that integrates exercise with patient education, aiming to surpass the benefits of exercise alone for individuals with prediabetes or diabetes in middle-income countries. Our objectives include improving functional capacity, increasing disease-related knowledge, fostering healthier behaviors, optimizing cardiometabolic health parameters, enhancing quality of life, alleviating depression, enhancing diet quality, and reducing diabetes morbidity over 6 months.

This study has the potential to contribute significantly by assessing the effectiveness of a structured patient education program associated with an exercise program that could be applied in the public and private health system in order to improve outcomes related to the prognosis in individuals with diabetes and to prevent type 2 diabetes in individuals with prediabetes. Considering (1) the proven benefits of physical exercise for individuals with diabetes and prediabetes, (2) the role of health education in facilitating better diabetes management and prevention, and (3) its impact on mitigating complications and cardiovascular risk factors, these interventions could substantially improve the quality of life for this population.

## Trial status

Recruitment began in November 2022 and ended in February 2024. The current protocol is version 9.0, dated on 26 June 2024. It is anticipated that data collection will be completed by December 2024, following the last participant’s completion. Submitting the study protocol earlier was not feasible due to challenges, including researcher turnover in the study research team and multiple updates in the study protocol due to the COVID-19 pandemic and the pilot [22] and feasibility [26] studies motivated by that. This trial closure is scheduled for May 2025, following the data analysis completion.

## Supplementary Information

Additional file 1. Screener for internet access and technology literacy.

Additional file 2.

## Data Availability

To ensure transparency in the study, the datasets and statistical analyses from this trial will be made available upon reasonable request from the corresponding author after the trial has been completed.

## Abbreviations

LMIC: Low- and middle-income countries
HIC: High-income countries
RCT: Randomized clinical trial
ExLE: Exercise and Lifestyle Education Program
Ex: Exercise Program
FFQ: Food frequency questionnaire
HRV: Heart rate variability
GEE: Generalized estimation equations
ISWT: Incremental shuttle walking test
DATE-Q: DiAbeTes Education Questionnaire
NVS: Newest vital sign
MDS: Mediterranean Diet Scale
SF-36: Medical Outcomes Study 36-Item Short-Form Health Survey
BESES: Bandura’s exercise self-efficacy scale
MAT ADOs and MAT insulin: Measure of Adherence to Oral Antidiabetic and Insulin Treatments questionnaire
A1c: Glycated hemoglobin
BMI: Body mass index
iNN: Normal RR intervals
SDNN: Standard deviation of normal RR intervals
RMSSD: Root mean square of the differences between successive normal RR intervals
pNN50: Percentage of successive normal RR intervals with a duration difference greater than 50 ms
SD1: Poincaré plot standard deviation perpendicular the line of identity
SD2: Poincaré plot standard deviation along the line of identity
LF: Low-frequency spectral component
HF: High-frequency spectral component
TP: Total power
