# Supplementary material for: Effects of an Exercise and Lifestyle Education Program in Brazilians living with prediabetes or diabetes: study protocol for a multicenter randomized controlled trial
Source: Trials. 2024 Oct 21;25:701. doi: 10.1186/s13063-024-08535-6 (PMC11492483; doi:10.1186/s13063-024-08535-6)
Supplement: Supplementary file 1 — Additional file 1. Screener for internet access and technology literacy. [file 13063_2024_8535_MOESM1_ESM.docx]

**Additional File 1**

SCREENER FOR INTERNET ACCESS AND TECHNOLOGY LITERACY.

| **Question** | **Yes** | **No** |
| --- | --- | --- |
| Do you have access to an internet-connected device (such as a smartphone, computer, tablet, or iPad)? |  |  |
| Do you have access to the WhatsApp Messenger? |  |  |
| Do you feel comfortable using the internet to send and receive messages via WhatsApp, browse a website, and watch videos? |  |  |
| Are you willing to participate in the study using the internet? |  |  |
